# p38 MAPK‐mediated loss of nuclear RNase III enzyme Drosha underlies amyloid beta‐induced neuronal stress in Alzheimer's disease

**DOI:** 10.1111/acel.13434

**Published:** 2021-09-16

**Authors:** Haidong Xu, Xiaolei Liu, Wenming Li, Ye Xi, Peng Su, Bo Meng, Xiaoyun Shao, Beisha Tang, Qian Yang, Zixu Mao

**Affiliations:** ^1^ Department of Pharmacology and Chemical Biology Emory University School of Medicine Atlanta Georgia USA; ^2^ Department of Neurology Xiangya Hospital Central South University Changsha China; ^3^ Department of Neurosurgery Tangdu Hospital The Fourth Military Medical University Xi'an China; ^4^ Department of Neurology Emory University School of Medicine Atlanta Georgia USA

**Keywords:** Alzheimer's disease, amyloid beta (Aβ), Drosha, neuronal death, p38 MAPK

## Abstract

MicroRNAs (miRNAs) are small noncoding RNAs ubiquitously expressed in the brain and regulate gene expression at the post‐transcriptional level. The nuclear RNase III enzyme Drosha initiates the maturation process of miRNAs in the nucleus. Strong evidence suggests that dysregulation of miRNAs is involved in many neurological disorders including Alzheimer's disease (AD). Dysfunction of miRNA biogenesis components may be involved in the processes of those diseases. However, the role of Drosha in AD remains unknown. By using immunohistochemistry, biochemistry, and subcellular fractionation methods, we show here that the level of Drosha protein was significantly lower in the postmortem brain of human AD patients as well as in the transgenic rat model of AD. Interestingly, Drosha level was specifically reduced in neurons of the cortex and hippocampus but not in the cerebellum in the AD brain samples. In primary cortical neurons, amyloid‐beta (Aβ) oligomers caused a p38 MAPK‐dependent phosphorylation of Drosha, leading to its redistribution from the nucleus to the cytoplasm and a decrease in its level. This loss of Drosha function preceded Aβ‐induced neuronal death. Importantly, inhibition of p38 MAPK activity or overexpression of Drosha protected neurons from Aβ oligomers‐induced apoptosis. Taken together, these results establish a role for p38 MAPK‐Drosha pathway in modulating neuronal viability under Aβ oligomers stress condition and implicate loss of Drosha as a key molecular change in the pathogenesis of AD.

## Graphical abstract

The nuclear RNase III enzyme Drosha was decreased in the postmortem brain of human AD patients as well as in TgF344‐AD rat. In primary cortical neurons, amyloid‐beta (Aβ) oligomers caused p38 MAPK‐dependent phosphorylation and reduction in Drosha, which preceded neuronal death. Our study identified loss of Drosha as a key molecular event in the pathogenesis of AD.

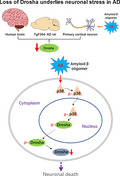

## 1 INTRODUCTION

Alzheimer's disease (AD) is hallmarked by amyloid plaques, tau neurofibrillary tangles, and progressive degeneration of neurons (Selkoe, 2011). Amyloid beta (Aβ), the principal component of amyloid plaque, is believed to act early in the disease to trigger the downstream pathogenic process (Hardy & Selkoe, 2002). Aβ is produced from the sequential cleavages of β‐amyloid precursor protein (APP) by β‐secretase and γ‐secretase enzymes. Soluble Aβ oligomers resulting from increased level of Aβ are now regarded as the main pathological species. Studies of genetic factors predisposing to the development of AD have identified genes encoding ApoE, presenilin 1 (PS1), presenilin 2 (PS2), and APP. It is well established that their mutations all lead to the accumulation of the toxic Aβ and cause AD (Selkoe, 2002, 2011). The processing of APP is subjected to complex regulation by many factors, which collectively determines the ultimate level of Aβ. Recent studies have revealed the emerging role of microRNAs (miRNAs), the small noncoding RNAs of about 22 nucleotides, in AD. Indeed, several miRNAs are found to regulate APP at post‐transcriptional level and their levels are abnormal in AD patients (Hebert et al., 2008; Smith et al., 2011), which might contribute to the accumulation of Aβ protein in the brain. Thus, dysregulation of miRNAs biogenesis might play an essential role in the pathogenesis of AD.

miRNAs regulate gene expression at the post‐transcriptional level through base pairing with their mRNA targets (Ha & Kim, 2014), thus effectively modulating the activity of more than half of human protein‐coding genes and function in almost all aspects of biological processes (Ha & Kim, 2014; Huntzinger & Izaurralde, 2011). miRNAs biogenesis are regulated by several tightly coupled steps (Lee et al., 2002). The transcription of primary miRNA (pri‐miRNA) is initially carried out by RNA polymerase II (Pol II). The nuclear RNase III enzyme Drosha, together with DGCR8 in a complex named microprocessor, initiates the maturation process by cleaving pri‐miRNA to pre‐miRNA in the nucleus (Gregory et al., 2004; Han et al., 2004). Pre‐miRNA is exported into the cytoplasm in an exportin 5 dependent process and further processed into mature miRNA by the second RNase III‐type endonuclease, Dicer (Hutvagner et al., 2001). Thus, Drosha controls the initial step of miRNA maturation (Lee et al., 2003).

miRNAs express throughout the brain. Mutations or deletions of miRNA biogenesis‐related proteins cause abnormal brain development or lethality during embryogenesis (Babiarz et al., 2011; Bernstein et al., 2003; Deshpande et al., 2005; Giraldez et al., 2005), highlighting the essential role of miRNAs in brain development. Thus, it is not surprised that miRNAs and proteins associated with its biogenesis are also found to play an important role in many neurodegenerative diseases. Indeed, increasing evidence now supports the notion that dysregulation of miRNAs contributes to the key disease processes involved in the neuronal disorders, such as AD, Parkinson's disease, Rett, and fragile X syndromes, as well as in schizophrenia, depression, and drug addiction (Hebert et al., 2008; Im & Kenny, 2012; Kim et al., 2007). However, there is no clear evidence to date to show whether Drosha is dysfunctional in AD and contributes to the pathogenesis of the disease.

Our previous study has demonstrated that p38 MAPK directly phosphorylates Drosha under stress and leads to its degradation by calpain. This precedes and triggers stress‐induced cell death (Yang et al., 2015). In the present study, we present clear evidence demonstrating the dysregulation of Drosha level and function in AD using the brains of postmortem AD patients and transgenic rat model of AD, and primary cortical neurons treated with Aβ oligomers. Our study reveals that a novel p38 MAPK‐Drosha pathway underlies neuronal survival in the pathogenesis of AD.

## 2 RESULTS

### 2.1 Drosha levels are decreased in the brain of AD patients

**FIGURE 1:**
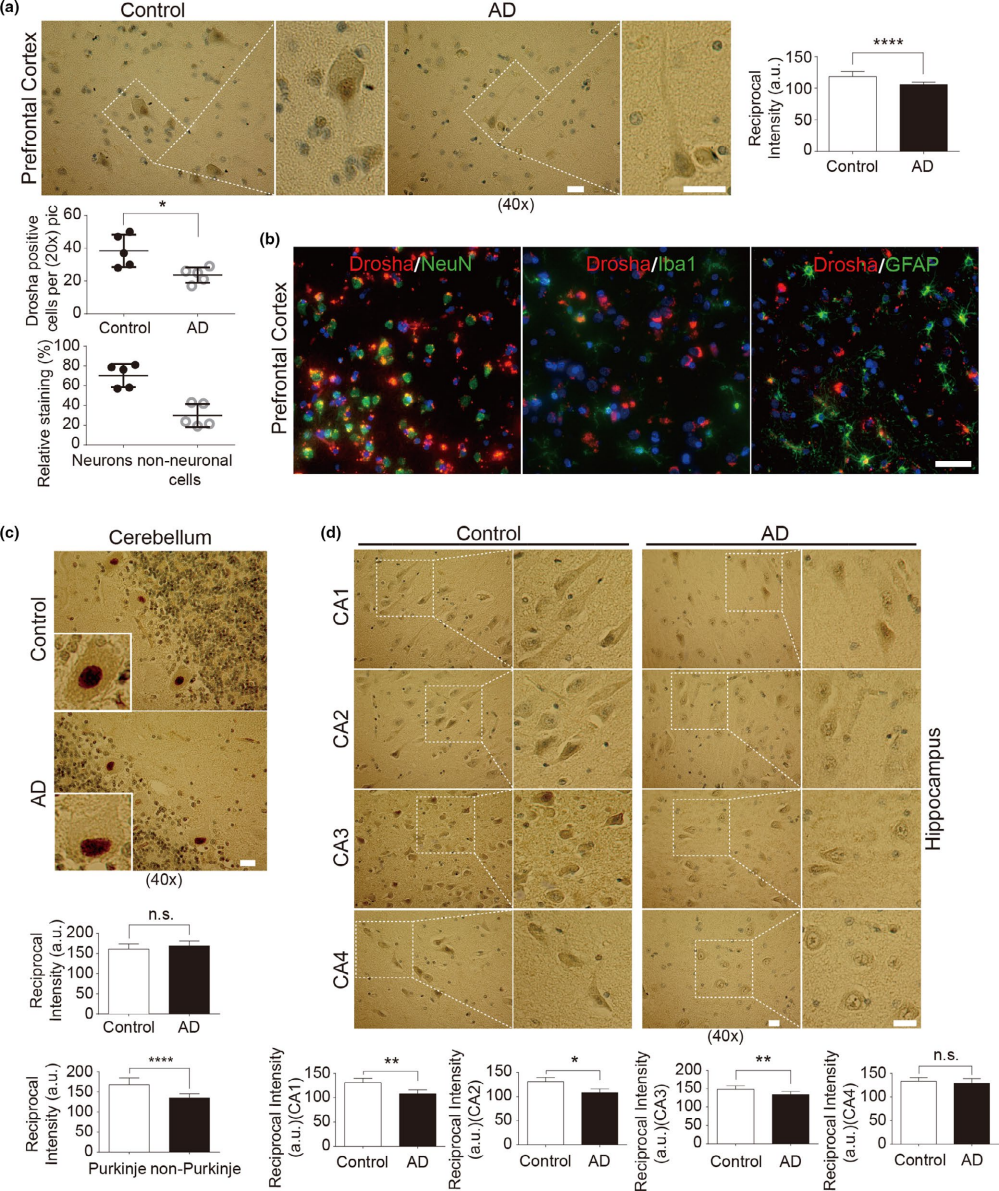
Drosha is decreased in the prefrontal cortex and hippocampus of AD patients. (a) Drosha immunohistochemistry in the prefrontal cortex. Areas in white boxes are shown at higher magnification on the right. The slices were counterstained with hematoxylin, and the nuclei number and Drosha‐positive cells (Figure S2a) were counted. The relative percentage staining in identified neurons and non‐neuronal cells in the prefrontal cortex of control was also quantified (at least 300 positive staining cells were counted). (b) Representative immunofluorescent images of prefrontal cortex slices of human control stained with Drosha and NeuN, or Iba1, or GFAP, respectively. Scale bar, 100 μm. (c–d) Drosha immunohistochemistry in the cerebellum (c) and hippocampus (d). Insets are enlarged images of a single cell (c) and areas in white boxes are shown at higher magnification on the right (d). The reciprocal intensities of DAB in each brain region as well as Drosha signals in Purkinje cells and other morphological non‐Purkinje cells (c) were analyzed by ImageJ (*n* = 5 control or AD cases for each area); Scale bar, 20 μm (a, c, and d). *p < 0.05, **p < 0.01, and ****p < 0.0001 versus control group. Error bars show mean ± SD

To investigate whether Drosha expression changes in the brains of human AD patients, we determined the level of Drosha in the postmortem brains of control and AD patients matched at age, gender, and postmortem interval by immunohistochemistry (IHC) (detail diagnostic information and statistical analysis are included in Tables S1 and S2). Among several anti‐Drosha antibodies from commercial sources (Table S3), we chose a mouse monoclonal antibody (Santa Cruz Biotechnology) for IHC staining and Western blot analysis based on our verification (Figure S1a). We confirmed its specificity by competition experiments using IHC (Figure S1b,c). We identified different cell types of the brain based on well‐established common morphological criteria including identifiable projections such as axons and dendrites and the large soma and nucleus for neurons as well as a smaller soma and nucleus and sometimes with many cell processes for glia. Based on such criteria, our IHC analysis showed that Drosha was positive in multiple cell types with the strongest signal from the cells with projections and relative larger soma and nuclei, which we identified as neurons (Figure 1a). It should be noted that Drosha signals were present predominantly in the nuclei while lower level of Drosha was also detected in the cytoplasm and projections (Figure 1a, enlarged images). Furthermore, our analysis revealed that both the intensity and number of Drosha‐positive cells were lower in AD than in the controls (Figure 1a, right and lower panels and Figure S2a). Quantification of Drosha‐positive cells with neuronal or non‐neuronal morphology showed that about 60%–80% staining for Drosha signal were from neurons in the deep layers of the prefrontal cortex analyzed (Figure 1a, lower panel). To corroborate the observation that most Drosha signals are from neurons, we stained human prefrontal cortex with antibodies to Drosha and different cell‐specific markers (NeuN, Iba1, or GFAP). This analysis showed that Drosha signal mainly colocalized with the neuronal marker NeuN and rarely with microglia‐specific marker, Iba1, and astrocyte‐specific marker, GFAP (Figure 1b). In the cerebellum, Drosha signal was strong in the nuclei of Purkinje cells with large cell body and nucleus in the control and AD samples (Figure 1c). Double staining of Drosha and Purkinje‐specific marker Calbindin confirmed that the strong signal of Drosha staining colocalized with Calbindin signal (Figure S2b), indicating that cells with strong signal of Drosha in Figure 1c were Purkinje neurons. Drosha signal in Purkinje neurons showed no significant difference between human control and AD patients (Figure 1c, lower panels). Analysis of the hippocampal tissue showed that Drosha was detectable in multiple cell types in various regions of the hippocampus but strongest in cells with pyramidal neuronal morphology with larger cell bodies and characteristic projections (Figure 1d). Drosha signal was more intensive in the nucleus than in the cytoplasm in the pyramidal neuron in controls. While it lost the strong nuclear staining and changed to a more diffused pattern throughout the nucleus and cytoplasm in AD brains (Figure 1d, enlarged images). This change was especially pronounced in the *Cornu Ammonis* (CA) 1, CA2, and CA3 regions (Figure 1d, lower panels). Together, these data indicate clearly that Drosha, although present in multiple types of human brain cells, is high in neurons and neuronal Drosha is reduced in the prefrontal cortex and hippocampus of AD patients.

**FIGURE 2:**
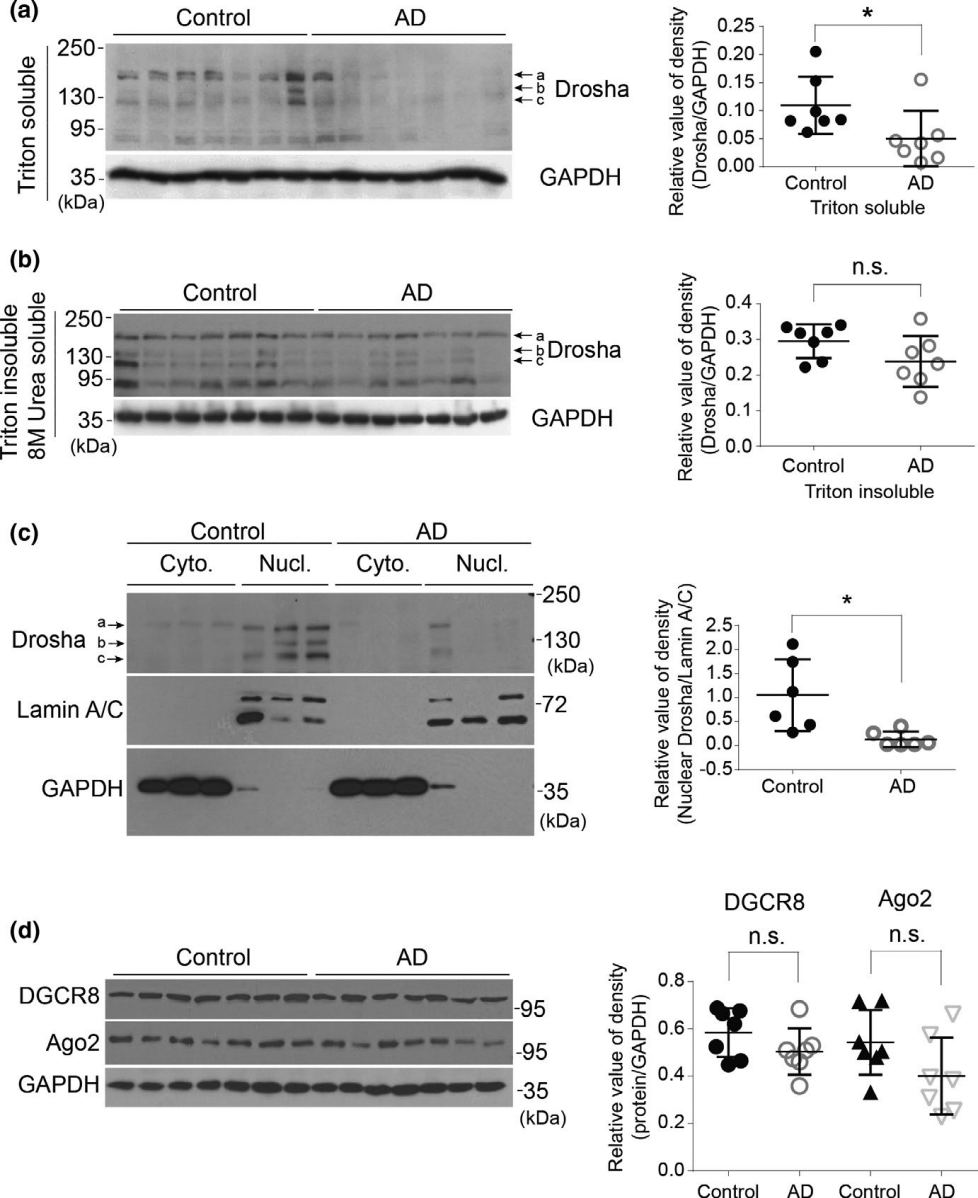
Soluble Drosha is decreased in the nucleus of human AD brain. (a–b) Triton X‐100 soluble and 8 M urea soluble (Triton insoluble) lysates were immunoblotted with Drosha antibody. GAPDH was used as a loading control. Right panels show quantitative analysis of the protein levels of Drosha (*n* = 7). (c) Cytosol and nuclear fractions were prepared from human prefrontal cortex and analyzed as shown (*n* = 6). (d) Triton soluble lysates prepared from human prefrontal cortex were blotted (*n* = 7). Data showed here are the representative blots from three independent experiments. *p < 0.05 and n.s. (not significant) versus the control groups. Error bars show mean ± SD

Next, we examined the protein level of Drosha in the prefrontal cortex of human brain by immunoblotting. Seven normal control and AD patients matched in age, sex, and postmortem interval (Tables S1 and S2) were chosen for the study. Analysis of the postmortem brain samples lysed with 1% Triton X‐100 buffer for Western blot revealed the presence of multiple forms of Drosha with different molecular mass (Drosha a, ~160 kDa, Drosha b, ~145 kDa, and Drosha c, ~125 kDa) in the control group, consistent with previous study (Gregory et al., 2004). Compared with the controls, the level of Triton X‐100 soluble Drosha was significantly lower in the AD brains (Figure 2a). Since the Triton X‐100 leaves significant cellular components including aggregated proteins, cytoskeleton, and insoluble membrane (London & Brown, 2000), we tested other extraction conditions such as high salt or urea. We found that 8 M urea extracted significant amount of Drosha from the Triton insoluble pellet. In contrast to Triton X‐100 soluble Drosha, the levels of Triton insoluble but 8 M urea soluble Drosha were not different between control and AD groups (Figure 2b). Since Drosha appears to be mainly expressed in neurons based on IHC staining (Figure 1), these data suggested that the decrease of Triton X‐100 soluble Drosha likely reflects a reduction in Drosha in neurons in the AD brains. As the main component of microprocessor, Drosha is mainly localized in the nucleus to process the pri‐miRNA to pre‐miRNA. To determine whether Drosha changes in the nucleus, we analyzed Triton X‐100 soluble Drosha in the cytosol and nuclear fractions prepared from postmortem brain samples. The results showed that the protein levels of Drosha in the nucleus were greatly reduced compared with that in the controls (Figure 2c). We did not detect significant change of other miRNA biogenesis or assembly‐related proteins, such as DGCR8 or Argonaute 2 (Ago2) between control and AD patients (Figure 2d). Considering that the staining for Drosha is stronger in the nuclei of neurons, these results indicate clearly that there is a significant decrease in the level of neuronal nuclear Drosha in the brains of AD patients.

### 2.2 Drosha levels are decreased in the brain of AD transgenic rats

**FIGURE 3:**
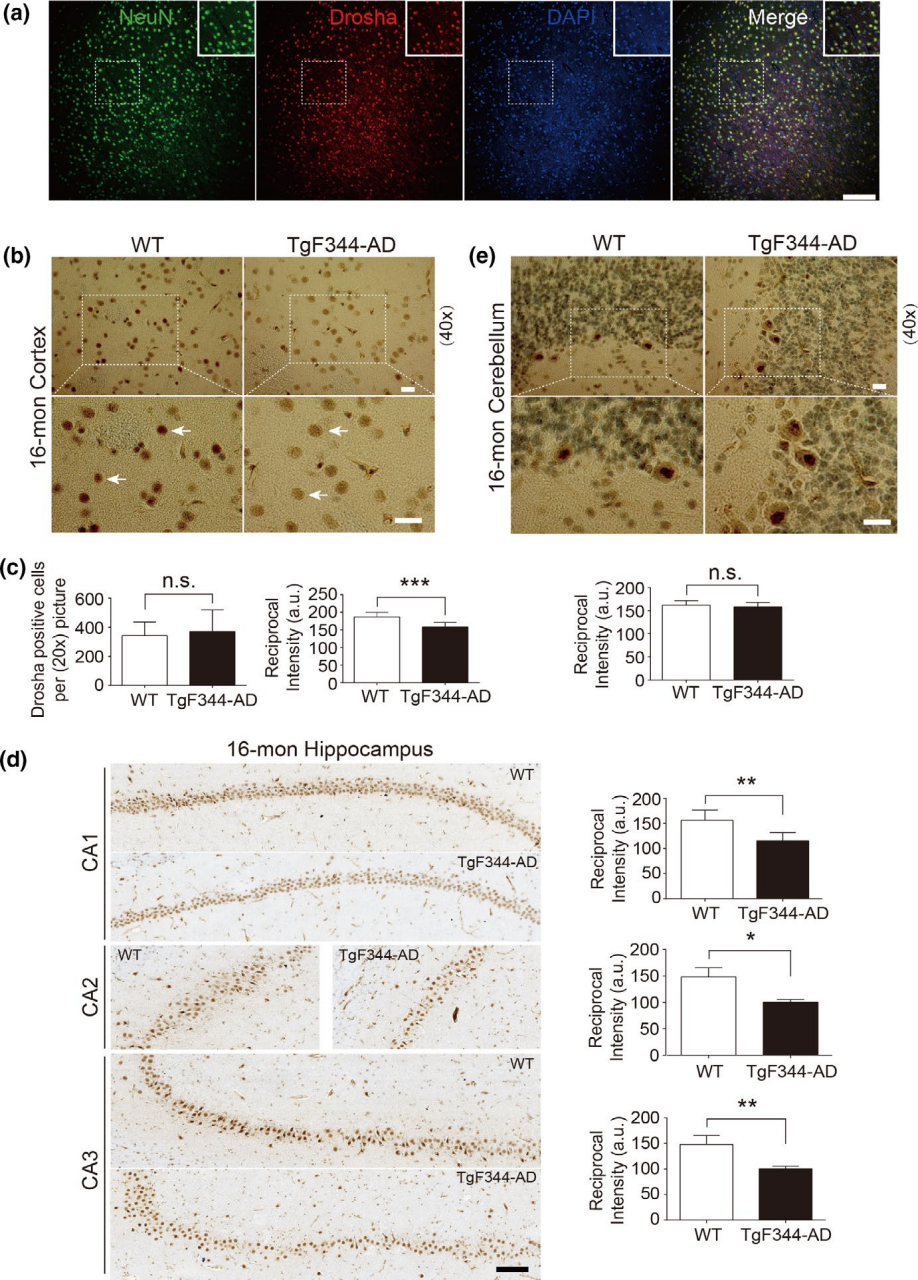
Drosha is decreased in the brains of transgenic rat model of AD, TgF344‐AD. (a) Representative immunofluorescent images of rat cortical brain slices stained with NeuN (green), Drosha (red), and DAPI (blue). (b–e) Drosha immunohistochemistry in the cortex (b), hippocampus (d), and cerebellum (e). Areas in white boxes are shown at higher magnification. Arrows (b) indicated Drosha staining in the nucleus. The number of Drosha‐positive cells and signal intensity in (b) were quantified (c). Scale bar, 200 μm (a), 20 μm (b and e), and 100 μm (d). *p < 0.05, **p < 0.01, ***p < 0.005, and n.s. (not significant) versus WT groups. Data were acquired from four animals (*n* = 4). Error bars show mean ± SD

**FIGURE 4:**
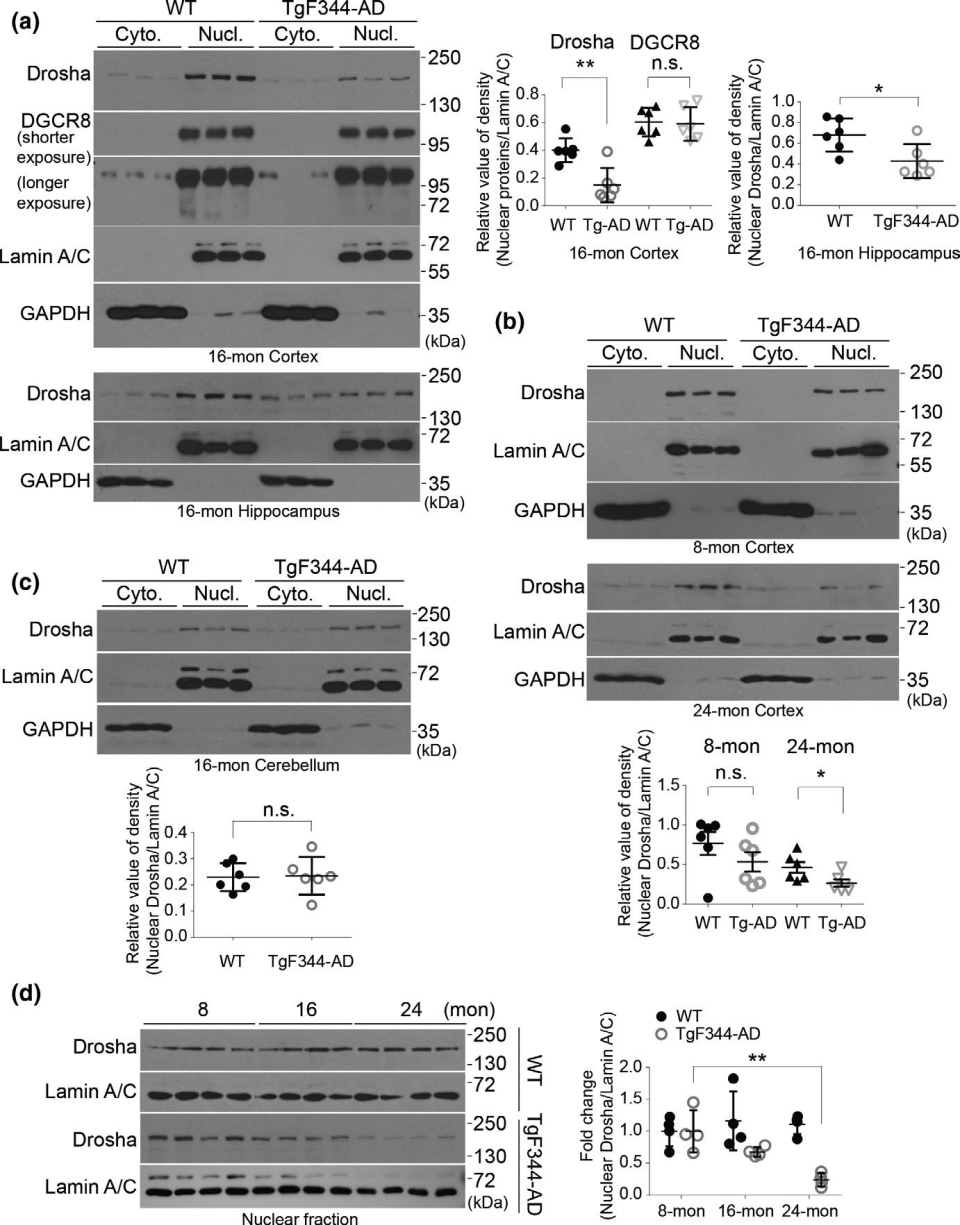
Nuclear Drosha is decreased in the brain of TgF344‐AD rat. (a–b) The protein levels of Drosha and DGCR8 in the cortex and hippocampus (a), as well as Drosha level in the cerebellum (b) of 16‐month WT and TgF344‐AD rats were examined by Western blot. Graphs are the quantitative analysis of the indicated proteins (*n* = 6). (c) The protein levels of Drosha in the cortex of 8‐ and 24‐month WT and TgF344‐AD rats were examined by Western blot. Quantitative analysis is shown below (*n* = 6). (d) Nuclear Drosha changes with aging. Levels of nuclear Drosha in the cortical tissue of animals at different ages were analyzed and quantified (*n* = 4). Data showed here are the representative blots from three independent experiments. *p < 0.05, **p < 0.01, and n.s. (not significant) versus WT groups. Error bars show mean ± SD

To corroborate with the findings from postmortem human brains, we assessed Drosha in a transgenic rat AD model (line TgF344‐AD), which expresses mutant human amyloid precursor protein (*APP_sw_*) and presenilin 1 (*PS1△E9*) genes (Cohen et al., 2013). TgF344‐AD rats manifest the full spectrum of AD pathologies including age‐dependent cerebral amyloidosis that precedes tauopathy, gliosis, apoptotic loss of neurons in the cerebral cortex and hippocampus, and cognitive disturbance (Cohen et al., 2013). As in human brain, neurons in rat brain also have large soma, astrocytes show a fibrous and stellate shape, and resting microglia have a small soma without extensive branches (Figure S2d). To confirm the identification of Drosha‐positive cells in rat brain, we stained the cortical brain slices for NeuN, GFAP, and Iba1, markers of neurons, astrocytes, and microglia, respectively, and Drosha. Double immunofluorescence showed that Drosha was expressed at high level in the nucleus in neurons, but its level was much lower in a small number of astrocytes (Figure 3a and Figure S2e). To determine whether Drosha changes in TgF344‐AD rats, we chose 16‐month‐old animals because at this age TgF344‐AD rats show significant changes in all major cytopathological features including Aβ deposition and neuronal loss (Cohen et al., 2013). Consistent with immunofluorescence study, IHC staining revealed that cortical Drosha was predominantly present in the nucleus (Figure 3b), similar to that in the human brain. Detailed analysis showed that although the number of Drosha‐positive cells in the cortex of WT and TgF344‐AD rats were similar, the intensity of Drosha signal was significantly reduced in the vast majority of cells in TgF344‐AD rats compared with those in WT rats (Figure 3c). In the CA and dentate gyrus (DG) areas of hippocampus, Drosha signal was high in the nucleus but also present in the cytoplasm of cells morphologically identified as pyramidal neurons in control rat (Figure 3d). Compared with WT rats, Drosha signals in the nuclei showed varying levels of significant reduction in the pyramidal neurons in the CA1‐3 areas of TgF344‐AD rats (Figure 3d). In contrast to cortical and hippocampal areas, Drosha staining in the cerebellum is predominant in cells identified as Purkinje neurons, and the staining was comparable between WT and TgF344‐AD rats (Figure 3e). Analysis of the cortical and hippocampal lysates from 16‐month‐old rats showed that Drosha was primarily present in the nuclear fraction and its level was significantly reduced in TgF344‐AD rats compared with control rats (Figure 4a). Furthermore, immunofluorescence analysis of 16‐month cortex of TgF344‐AD rats found no clear correlation of the levels of Drosha and distance to Aβ deposition (Figure S3a). Given our findings in Figure 3, we believed that the decrease of Drosha detected by Western blot in TgF344‐AD rat brain lysates mostly likely resulted from loss of Drosha in the neuronal nuclei. The levels of Drosha in the cerebella were similar between control and TgF344‐AD rats (Figure 4c). Furthermore, the levels of DGCR8 were also similar in the cortex between the two groups of rats (Figure 4a). Previous study showed that from 6 to 26 months (6, 16, and 26) TgF344‐AD rats showed progressive changes of Aβ deposition/oligomer starting at 6 months (Cohen et al., 2013). We reasoned that Drosha level may change in an aging‐dependent manner and thus determined Drosha levels in different ages of animals. Interestingly, while, as for 16‐month samples, the nuclear Drosha was much lower in 24‐month TgF344‐AD cortical samples than in WT brains, the level of nuclear Drosha had a decreasing tendency but was comparable at 8‐month between WT and TgF344‐AD cortical lysates (Figure 4b). We compared the levels of Drosha during aging. The results showed that the level of the nuclear Drosha did not change significantly in the cortex between 8‐, 16‐, and 24‐month WT rats. In contrast, nuclear Drosha decreased significantly over time in TgF344‐AD rats (Figure 4d). These results demonstrate clearly an age‐dependent loss of Drosha in the neuronal nuclei of TgF344‐AD rat brain.

### 2.3 Amyloid‐beta oligomers decrease Drosha and impair microprocessor cleavage activity

**FIGURE 5:**
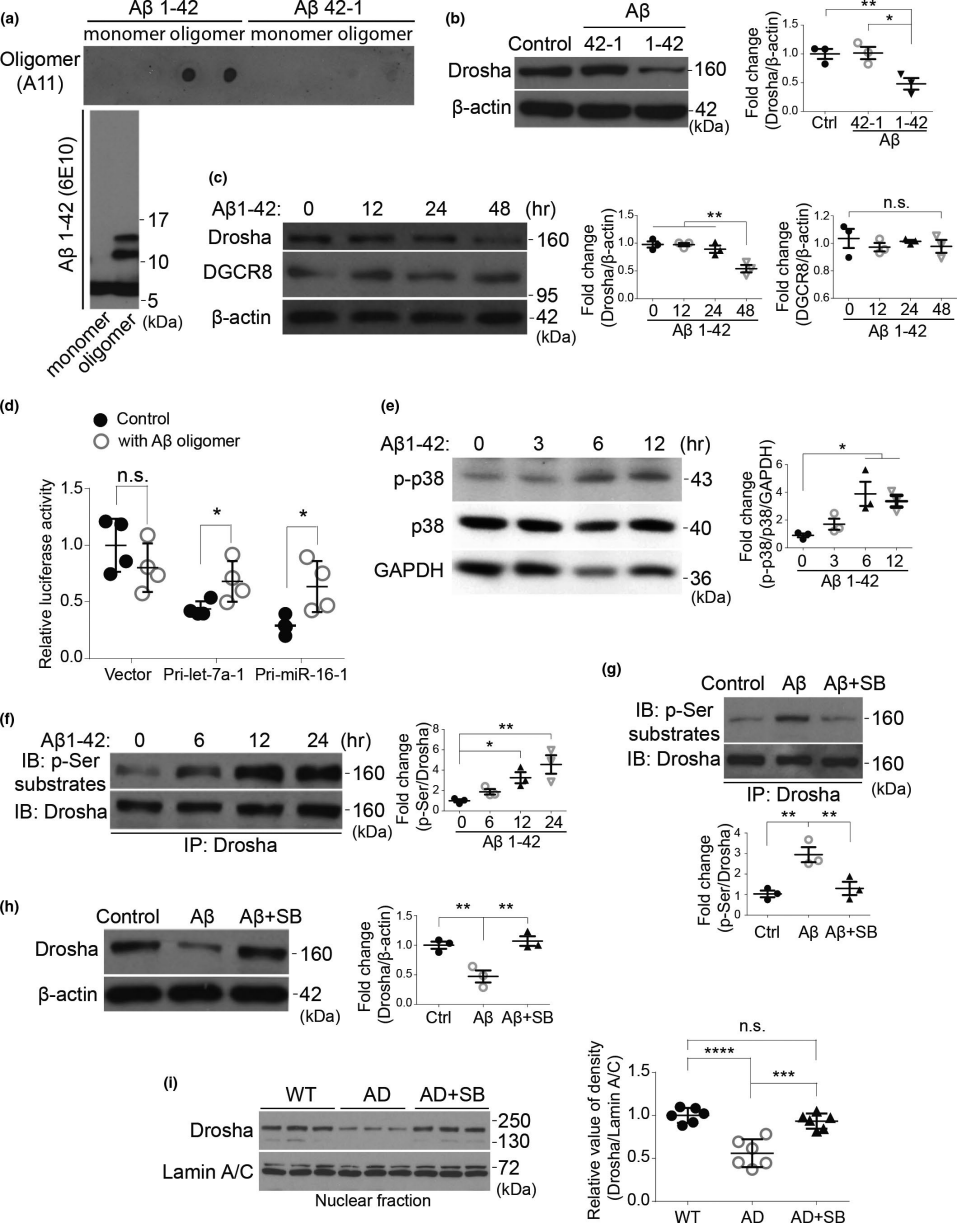
Aβ oligomers reduce Drosha level in a p38 MAPK‐dependent manner in primary cortical neurons. (a) Aβ oligomers were confirmed by dot blot with A11 antibody and by Western blot with 6E10 antibody. (b) Rat primary cortical neurons at 14 DIV were treated with 1 μM of Aβ 1‐42 or 42‐1 oligomers for 36 h, and the protein levels of Drosha and β‐actin were examined by Western blot and quantified. (c) Rat primary cortical neurons at 14 DIV were treated with 1 μM of Aβ 1‐42 oligomers for the indicated time, and the protein levels of Drosha, DGCR8, and β‐actin were examined by Western blot and quantified on the right. (d) Rat primary cortical neurons at 14 DIV were transfected with empty vector control or Pri‐let‐7a‐1 or Pri‐miR‐16‐1 reporters for 24 h and treated with 1 μM of Aβ 1‐42 oligomers for another 24 h. The dual‐luciferase assays were performed. RL‐luciferase activities were normalized with FF‐luciferase, and the percentage of relative enzyme activity compared with the control (vector reporter) was plotted. Error bars represent mean ± SD from four replicates. (e) Rat primary cortical neurons were treated with Aβ 1‐42 oligomers (1 μM) for the indicated time, and the phosphorylated p38, p38, and GAPDH were blotted and quantified. (f) Primary cortical neurons were treated with Aβ oligomers for the indicated time and Drosha was immunoprecipitated with anti‐Drosha antibody and the phosphorylated Drosha was examined with phospho‐Ser substrate antibody and quantified. (g) Primary cortical neurons were treated with Aβ 1‐42 oligomers (1 μM), or Aβ 1‐42 oligomers with SB203580 (10 μM) for 12 h. Drosha immunoprecipitated from lysates was blotted with the anti‐phospho‐Ser antibody and quantified below. (h) Drosha level from the primary cortical neurons treated with Aβ 1‐42 oligomers (1 μM) for 36 h in the presence of SB203580 or not was blotted and quantified. (*n* = 3 for b–h). (i) Twelve‐month‐old WT or TgF344‐AD rats were injected intraperitoneally (i. p.) with either DMSO or SB203580 (2 μg/g body weight). After three days, the nuclear fraction prepared from the cortex was blotted and quantified on the right (*n* = 6). Data showed here are the representative blots from at least three independent experiments. *p < 0.05, **p < 0.01, ***p < 0.005, ****p < 0.0001, and n.s. (not significant) versus the indicated groups. Error bars show mean ± SD

Downregulation of Drosha in human AD patients and in *APP*/*PS1* transgenic rat prompted us to test whether toxic stress associated with AD pathogenesis is involved in downregulation of Drosha. To investigate the possible mechanism, Aβ oligomers prepared from synthesized human toxic Aβ (1‐42) were confirmed by dot blot using Aβ oligomers‐specific antibody A11 and immunoblot with 6E10 antibody (Figure 5a). Aβ 1‐42 oligomers but not 42‐1 control peptides caused a significant decrease of Drosha in the primary cortical neurons (Figure 5b). Furthermore, the decrease in Drosha was time dependent. In contrast, the level of DGCR8 was not affected (Figure 5c). Together, these findings indicate that toxic Aβ peptide triggers a specific dysregulation of Drosha in those neurons. To test whether Aβ oligomers affect microprocessor cleavage activity, we transfected neurons separately with two pri‐miRNA luciferase reporters whose values are sensitive to and negatively correlated with microprocessor's ability of converting pri‐ to pre‐miRNA (Dai et al., 2016) and treated the cells with Aβ oligomers. Under the condition with normal basal Drosha activity, the value of either pri‐miR‐16‐1 or pri‐let‐7a‐1 reporter was lowered compared to that in the control group (Figure 5d). Aβ oligomers treatment drastically increased the level of both reporters, indicating that the microprocessor cleavage activity was impaired. As Aβ oligomers decreased Drosha level without affecting DGCR8, these data suggest that Aβ oligomers selectively impair Drosha of microprocessor.

### 2.4 Amyloid‐beta oligomers decrease Drosha in a p38 MAPK‐dependent manner

Our recent study revealed that Drosha is targeted for degradation under stress conditions through a mechanism involving p38 MAPK‐dependent phosphorylation of Drosha (Yang et al., 2015). To explore whether p38 MAPK was involved in the downregulation of Drosha upon Aβ oligomers treatment, we first examined whether p38 MAPK was activated by Aβ. This analysis showed that Aβ 1‐42 oligomers caused time‐dependent and persistent increase in p38 phosphorylation (Figure 5e). To examine whether Drosha were phosphorylated by p38 upon Aβ oligomers challenge, we immunoprecipitated endogenous Drosha and blotted with an antibody specifically recognizes proline‐directed phosphorylated serine. Results showed that Aβ treatment led to a time‐dependent upregulation of Drosha phosphorylation (Figure 5f), which was clearly reduced by p38 MAPK inhibitor SB203580 (Figure 5g). p38 MAPK inhibitor SB203580 also greatly attenuated Aβ‐induced loss of Drosha (Figure 5h). Since Drosha level was reduced in TgF344‐AD rats as early as 12 months of age, we performed *in vivo* pharmacological intervention using this age of animals. *In vivo* administration of SB203580 significantly rescued the nuclear Drosha in 12 months TgF344‐AD rats to a level comparable to that in WT rats (Figure 5i). These data suggest that Aβ oligomers induce a p38 MAPK‐dependent phosphorylation and this may be responsible for loss of Drosha in primary cortical neurons and TgF344‐AD rats.

### 2.5 Amyloid‐beta peptides decrease Drosha level in neurons and overexpression of Drosha protects neurons from amyloid‐beta peptides‐induced toxicity

**FIGURE 6:**
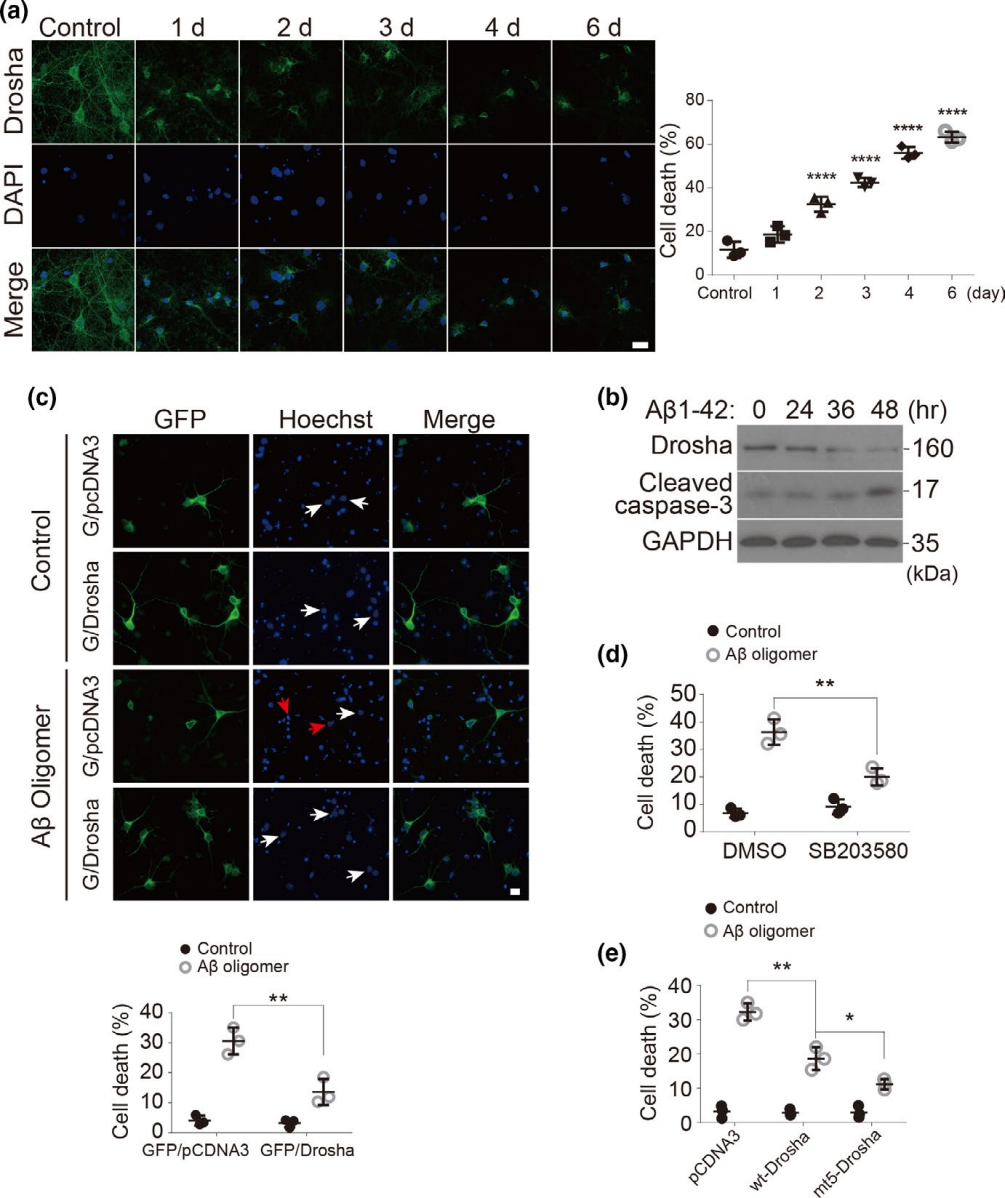
Aβ oligomers decrease Drosha levels and induce apoptosis in neurons and Drosha overexpression prevents Aβ oligomers‐induced neuronal death. (a) Primary cortical neurons at 14 DIV were treated with Aβ 1‐42 oligomers (1 μM) or its control (Aβ 42‐1) for 1–6 days. The neurons were stained with Drosha and DAPI for fluorescence microscopy analysis. Scale bar, 20 μm. Quantitative analysis of the neurons with condensed or fragmented nuclei (over 200 neurons were counted, *n* = 3). (b) Rat primary cortical neurons at 14 DIV were treated with Aβ 1‐42 oligomers (1 μM) for the indicated time and the levels of Drosha, cleaved caspase‐3 and GAPDH were blotted. Data showed here are the representative one from three independent experiments. (c) Primary cortical neurons at 14 DIV were co‐transfected with GFP/pcDNA3 or GFP/Drosha for 12 h and treated with Aβ 1‐42 oligomers (1 μM) or its control (Aβ 42‐1) for 48 h. The neurons were stained with Hoechst and fixed with paraformaldehyde for fluorescence microscopy analysis. The white arrows indicate the nucleus of neurons transfected with plasmids, and the red arrows indicate the abnormal nuclear morphology of transfected neurons. Quantitative analysis of the transfected neurons with condensed nuclei was shown below (over 300 neurons were counted). Scale bar, 5 μm. (d) Primary cortical neurons were treated with Aβ 1‐42 oligomers (1 μM) for 48 h in the presence of SB203580 (10 μM) or not, and the neuronal death based on nuclear morphology were performed and quantified. (e) Primary cortical neurons at 14 DIV were transfected with pcDNA3, wt‐Drosha, or mt‐Drosha (five putative p38 phosphorylation sites mutated to alanine) for 12 h and treated with Aβ 1‐42 oligomers or its control (Aβ 42‐1) for another 48 h. Neuronal death based on nuclear morphology were performed and quantified. (*n* = 3 for d, e). Data showed here are the representative one from three independent experiments. Quantitative analysis of the neuronal death based on nuclear morphology were performed as in (c) *p < 0.05, **p < 0.01, and ****p < 0.0001 versus the indicated groups

The accumulation of Amyloid‐β peptides, especially soluble oligomeric species, has been implicated in triggering downstream pathogenic processes including tau pathology and neuronal loss in AD (Long & Holtzman, 2019) while Drosha activity appears to be required for cell survival under stress conditions (Yang et al., 2015). We first characterized the change of endogenous Drosha in primary cortical neurons treated with Aβ oligomers for 1–6 days. Drosha was present in the nuclei as well as in the soma, and processes under normal culture condition. Aβ treatment for 1 days led to the redistribution of nuclear Drosha to and accumulation in the soma (Figure 6a). This redistribution continued and was companied with significant loss of Drosha starting at 36 h, which preceded a significant increase in activated caspase‐3 at day 2 (Figure 6a,b). Extended Aβ oligomers treatment for 3–6 days significantly decreased the Drosha levels in most neurons and resulted in a progressive increase in neuronal death measured by nuclear chromosome condensation or fragmentation (Figure 6a). The finding that loss of Drosha precedes caspase‐3 activation and loss of neuronal viability indicates that loss of Drosha trigger the subsequent apoptosis. This led us to investigate whether Drosha overexpression could protect the neurons from Aβ oligomers‐induced toxicity. We showed first that Flag‐Drosha after transfection of primary cortical neurons (efficiency estimated to vary between 7% and 15%) was present predominantly in the nucleus but also in the cytoplasmic compartment (Figure S3b). Primary cortical neurons were co‐transfected with GFP and pcDNA3, or GFP and pcDNA3‐Drosha plasmids, and then were treated with Aβ 1‐42 oligomers or Aβ 42‐1, respectively. Results showed that GFP or Drosha alone did not significantly affect the nuclear morphology of neurons (Figure 6c, white arrows in control panels). Aβ oligomers treatment induced a significant increase in apoptosis in the GFP/pcDNA3 group (Figure 6c, red arrows in the upper panel of Aβ oligomers treatment). However, Drosha overexpression greatly blocked Aβ oligomers‐induced nuclear change (Figure 6c). These results indicate that Drosha effectively protects neurons from Aβ oligomers‐induced toxicity. Furthermore, p38 inhibitor partially blocked Aβ oligomers‐induced toxicity in neurons, indicating that p38 activation is involved this process (Figure 6d). To determine whether p38‐induced phosphorylation of Drosha is critical for Drosha‐mediated neuronal protection, we expressed either wild‐type Drosha (wt‐Drosha) or Drosha mutant which is resistant to p38 phosphorylation (mt5‐Drosha) and tested their potency in protecting cortical neuron against Aβ oligomers challenge. While wt‐Drosha offered protection, mt5‐Drosha was much more effective in protecting cortical neurons from Aβ oligomers‐induced toxicity (Figure 6e). Those data demonstrate that loss of Drosha may underlie Aβ oligomers‐induced neuronal death.

## 3 DISCUSSION

Amyloid‐β peptide was identified as the main components of meningovascular amyloid and amyloid plaques (Glenner & Wong, 1984; Masters et al., 1985). Aβ aggregation triggers the downstream disease processes including tau pathology and neurodegeneration (Long & Holtzman, 2019; Musiek & Holtzman, 2015). Despite intensive studies, the mechanisms by which Aβ leads to the pathogenesis of AD remain to be fully clarified. In this study, we found that AD associated pathogenic conditions trigger the dysregulation of Drosha in both *in vitro* and *in vivo* models of AD and postmortem brains of AD patients. This involves p38 MAPK‐dependent phosphorylation and destabilization of Drosha and loss of Drosha underlies Aβ‐induced neuronal death. Thus, our study identifies targeting Drosha as a critical mechanism mediating Aβ toxicity in the pathogenic process of AD.

Although Drosha is commonly believed to be expressed by all types of cells in the brain, it is not clear if its levels are different among various brain cells. Our IHC and immunofluorescence data from human and rat brain revealed that Drosha staining was stronger in neurons than in glial cells. The significance of this difference is not clear. It is possible that neurons require much robust capacity of miRNA biogenesis. Consistent with this, Drosha has been shown to be required for the generation of new neurons, but not astrocytes, in the adult mouse hippocampus (Pons‐Espinal et al., 2017). Knockout of Drosha activates oligodendrogenesis and reduces neurogenesis in adult dentate gyrus neuron stem cells (Rolando et al., 2016). In addition, Drosha appears to have neuronal functions independent of canonical miRNA biogenesis. For example, Drosha can regulate neurogenesis by destabilizing of Neurog2 miRNAs (Knuckles et al., 2012). Thus, neurons may require high level of Drosha to perform both canonical and non‐canonical functions.

The cortex and hippocampus are brain areas vulnerable in AD while the cerebellum is relatively spared from classical AD pathology. Consistently, AD‐like pathological changes mainly occur in the hippocampal and cortical regions but not cerebellum in the TgF344‐AD rats compared with the corresponding wild‐type rats. Therefore, the cerebellum can be used as a brain region control. Our data show that the level of Drosha is reduced in the cortex and hippocampus but not in the cerebellum of brains from human patients and a transgenic rat model of AD. These findings suggest that the loss of Drosha is highly selective and tightly correlated with the vulnerability in AD. In the hippocampus, we found that Drosha is reduced in the hippocampus of AD patients by IHC and in TgF344‐AD rats by both IHC and WB. Notably, we found that Drosha is significantly decreased in hippocampal CA regions of human AD patients and TgF344‐AD rats, including CA3, which plays a specific role in memory processes and neurodegeneration (Cherubini & Miles, 2015). Whether this reflects differences in species or stages of pathogenic process remains to be clarified. The strong straining of Drosha in the pyramidal neurons in the CA3 region indicates the potential role of Drosha in the hippocampal function. Indeed, the dysregulation of certain miRNAs in the hippocampus has been reported to contribute to the cognitive disturbances linked to AD (Flight, 2011; Zovoilis et al., 2011). Deficiency of DGCR8, the other essential component of microprocessor also leads to the abnormal processing of specific brain miRNA and working memory deficits (Fenelon et al., 2011). Thus, it is possible that downregulation of Drosha in CA3 may contribute to the cognitive impairment in AD.

Drosha initiates the maturation process in the nucleus (Gregory et al., 2004; Han et al., 2004). Our IHC results and immunoblot analysis of nuclear versus non‐nuclear fractions show that while most of Drosha is in the nucleus, there is also low level of Drosha staining in the cytoplasm. This cytoplasmic Drosha varies between brain regions. Consistently, our results showed that clear Drosha staining is present in the soma and dendrites of primary cortical neurons. Drosha has been reported to function in the cytoplasm. For example, a truncated Drosha without the nuclear localization signal (NLS) at its N‐terminus recently has been shown to reside in the cytoplasm and cleave pri‐miRNA effectively (Dai et al., 2016). Previous study showed that RNA virus causes Drosha to relocate from the nucleus to the cytoplasm and process cytoplasmic restricted pri‐RNAs (Shapiro et al., 2012). Together, these data strongly suggest that Drosha is present and functions both in and outside the nucleus in neurons. Interestingly, it is known that stress causes Drosha to be transported out of the nucleus (Yang et al., 2015). Consistent with this, we showed that Aβ oligomers also triggered nuclear to cytoplasmic redistribution of Drosha in neurons. Since this is usually coupled to increased degradation of Drosha, our current assumption is that this transient redistribution should not increase Drosha's cytoplasmic functions. Experimental clarification of this issue should further our understanding of the regulation and role of Drosha in the neuronal cytoplasm.

Our results indicate that Drosha in human brain is partitioned into different pools based on its solubility. At present, at least two pools of Drosha appear to exit, one Triton X‐100 soluble and the other Triton X‐100 insoluble but urea soluble. Interestingly, it is the Triton X‐100 soluble fraction of Drosha that shows a significant decrease in AD while the urea soluble Drosha remains unchanged between control and AD cases, suggesting that loss of specific pool of but not total Drosha may be more tightly correlated to AD condition. Drosha is known to form dimer and be associated with large complexes (Gregory et al., 2004; Han et al., 2004). It is worth noting that a recent study has identified the presence of Drosha in the inclusions in ALS patients with *c9orf72* mutation (Porta et al., 2015). Since the study (Porta et al., 2015) has noted a lack of clear evidence for the presence of Drosha aggregates in human AD brain, we think that at least the majority of the Triton X‐100 insoluble Drosha is unlikely caused by or associated with the pathological aggregates typically found in AD. Thus, the state of Drosha in the Triton X‐100 insoluble fraction and the mechanism that regulates the Drosha partition between these pools under basal and AD pathogenic conditions are important questions worth further investigation.

Our previous study show that several stress conditions destabilize Drosha by p38 MAPK‐mediated phosphorylation, which promotes Drosha nuclear export and degradation (Yang et al., 2015). p38 MAPK has been reported to be activated in early stages in AD (Sun et al., 2003), and MAPKs have long been viewed as therapeutic targets for neurodegeneration (Harper & Wilkie, 2003). The present study identifies that Aβ oligomers stress engages p38 MAPK to target Drosha, causing its redistribution from nucleus to cytoplasm and reducing its level and function. Inhibition of p38 MAPK reduces Drosha phosphorylation in neurons and significantly rescues nuclear Drosha level in TgF344‐AD rat. Importantly, inhibition of p38 MAPK or expression of non‐phosphorylable mt5‐Drosha protected cortical neuron from Aβ oligomers‐induced damage. These data support the notion that the p38 MAPK‐Drosha pathway is involved in the pathogenesis of AD. In addition, loss of Drosha precedes activation of caspase‐3 triggered by Aβ oligomers in cultured neurons and occurs in 8‐month TgF344‐AD rats before the reported significant neuronal loss at 16 month (Cohen et al., 2013) while overexpression of Drosha protects neurons from Aβ oligomers‐induced toxicity. They strongly support the notion that downregulation of Drosha is an early molecular event in pathogenesis and compromises neuronal viability in AD. Recently, dysfunction of Drosha has been reported to participate in several neuronal diseases, including spinal muscular atrophy (SMA) (Goncalves et al., 2018) and Parkinson's disease (Pignataro et al., 2017; Wang et al., 2018). Whether Drosha dysfunction may be involved in the pathogenesis of other neurodegenerative disorders remains to be established.

The current study used multiple models to elucidate the regulation and role of Drosha in the context of AD since each model has its advantages and limitations. Treatment of primary cortical neurons with Aβ oligomers represents a rather acute model *in vitro* while the TgF344‐AD rats overexpress APP/PS1 *in vivo* for a relatively long time. Samples from AD patients provide a snapshot for a chronic disease. Although there is no strict temporal comparison among those three models, we found that Drosha level decreased in all these samples. The phenomena observed in the brain of AD patients at the final stage of the disease provide limited mechanistic insight. We thus explored Aβ oligomers treatment of primary cortical neurons as a cellular model to investigate the potential mechanism. Aβ oligomers cause many changes in primary cortical neurons such as impairment of long‐term potentiation (Ronicke et al., 2011), impairment of axonal BDNF retrograde trafficking (Poon et al., 2011), and depleting ER calcium levels (Resende et al., 2008). It is well documented that Aβ dysregulates p38 MAPK (Criscuolo et al., 2015; Valles et al., 2008). Considering the difference between the *in vitro* primary cortical neuron model and the samples of AD patients, we verified some of the key mechanistic findings including the role of p38 MAPK in TgF344‐AD rat model. Importantly, given that inhibition of p38 MAPK significantly rescued the nuclear Drosha level in AD rats, these data confirm that p38 MAPK mediates the downregulation of nuclear Drosha in both *in vitro* and *in vivo* AD models.

The precise mechanisms of how Drosha exerts its protective role in AD models are not clear. It is likely that Drosha offers neurons protection via regulating miRNA biogenesis. Consistent with this possibility, it has been reported that miRNAs targeting genes involved in APP processing, such as BACE1/β‐secretase (Boissonneault et al., 2009; Hebert et al., 2008) are reduced in AD. APP (Vilardo et al., 2010) and tau (Dickson et al., 2013) themselves are also targeted by miRNAs. Thus, it is reasonable that part of Drosha's protective effects comes from its canonical function. Recent studies reveal that Dicer, which functions downstream of Drosha in miRNA biogenesis cascade, has a protective role in dopamine neurons (Chmielarz et al., 2017) and conditional knockout of Dicer results in tau hyperphosphorylation and neurodegeneration that resembles the pathological changes observed in AD (Hebert et al., 2010). Together, they highlight the importance of maintaining miRNA homeostasis in neurons. It should be noted that Drosha has been reported to have non‐canonical functions independent of miRNA biogenesis including transcriptional regulation and maintenance of genome integrity (Burger & Gullerova, 2015; Knuckles et al., 2012; Pong & Gullerova, 2018). Whether Drosha protects neurons through its non‐canonical functions and its role in AD pathogenesis requires further investigation.

## 4 EXPERIMENTAL PROCEDURES

### 4.1 Animals and *in vivo* experiment

TgF344‐AD rats heterozygous for an *APP_sw_*/*PS1△E9* transgene and wild‐type littermates were housed in the facility of Division of Animal Resources at Emory University. All animal procedures were performed under the Institutional Animal Care and Use Committee (IACUC) of Emory University compliance. No gender differences on any of the measures reported, and thus, males and females were combined for all analyses (Cohen et al., 2013). Rats were maintained on a 12 h light/dark cycle and give *ad libitum* access to food and water. For in vivo p38 inhibition experiment, 12‐month‐old AD male rats were injected intraperitoneally (i.p.) once a day with DMSO or SB203580 (2 μg/g body weight), respectively, for three days. The rats were euthanized at the fourth day, and the brain were dissected for further usage.

### 4.2 Patient cases

All brain tissues were obtained from the Brain Bank of the Emory Alzheimer's Disease Research Center (ADRC). The Institutional Review Board (IRB) of Emory University approved all procedures, and all subjects or the family of the deceased subjects' content was obtained according to the Declaration of Helsinki. The control and AD cases were matched with regard to age, race, gender, and postmortem interval. They were not diagnosed with other neurodegenerative diseases, including Parkinson's disease. The detailed information for diagnosis and statistical analysis are shown in Tables S1 and S2.

### 4.3 Plasmids and antibodies

GFP‐C2 plasmid was from Addgene. Drosha‐Flag and mt‐Drosha (Drosha with five putative p38 sites mutated to alanine) plasmids were previously described (Yang et al., 2015). Luciferase‐based Drosha cleavage reporters (Vector, Pri‐miR‐16‐1 and Pri‐let‐7a‐1) were kind gifts from Dr. Shuo Gu (National Cancer Institute, National Institutes of Health). These reporters were generated based on the psiCHECK‐2 vector (Promega, Madison, WI, USA) (Dai et al., 2016). Antibodies to Drosha (C‐7, sc‐393591) and Lamin A/C (E‐1, sc‐376248) were purchased from Santa Cruz Biotechnology (Dallas, TX, USA). Antibodies to DGCR8 were from Proteintech (Rosemont, IL, USA). Antibodies to NeuN (A60), GFAP, and Iba1 were purchased from Millipore (Billerica, MA, USA). Antibodies to phospho p38 (9211), phospho S/P (2325), GAPDH (5174), amyloid‐β (2454), and Calbindin (13176) were from Cell Signaling Technology (Danvers, MA, USA), and anti‐p38 antibody was purchased from BD Transduction Laboratories (San Jose, CA, USA). Anti‐Aβ (6E10) was from BioLegend (San Diego, CA, USA), and oligomer A11 antibody was from Thermo Fisher Scientific (Waltham, MA, USA). Anti‐Argonaute 2, anti‐Flag, and anti‐β‐actin were from Sigma, and anti‐mouse‐IgG was from Jackson ImmunoResearch Laboratories (West Grove, PA, USA).

### 4.4 Primary cortical neuron cultures

Culture of primary cortical neurons from Long Evans rats at embryonic day 18 was carried out as described previously (Mao & Wiedmann, 1999). Briefly, cerebral cortex of rat embryo was separated under microscope and cortical neurons were digested with 0.125% trypsin and plated on poly‐l‐lysine‐coated plates with Neurobasal medium containing 2% B‐27 and 0.5 mM glutamine (all from Invitrogen, Carlsbad, CA, USA). Cortical neurons were cultured to 14 days in vitro (DIV) before usage. All procedures were approved by the Institutional Animal Care and Use Committee of Emory University.

### 4.5 Immunohistochemistry

Human and rat paraffin‐embedded sections were deparaffinized by incubation at 56℃ overnight followed by immersion in xylene and hydrated in graded ethanol solutions. After washed with tap water, antigen retrieval was performed using Antigen Unmasking Solution (Vector Laboratories, Burlingame, CA, USA) by microwaving for 7 min at 99℃. Sections were allowed to cool at room temperature (RT) and rinsed with Tris‐Brij buffer (100 mM Tris‐Cl pH 7.5, 100 mM NaCl, 5 mM MgCl_2_, 0.075% Brij 35) for 3 × 5 min. Sections were blocked with blocking buffer (2% goat serum in Tris‐Brij) for 2 h at RT followed by washing with Tris‐Brij 3 × 5 min and then incubated with anti‐Drosha antibody (1:300, diluted in blocking buffer) overnight at 4℃. On the second day, the sections were washed with Tris‐Brij 3 × 5 min and the endogenous peroxidases were quenched with 3% H_2_O_2_/methanol for 15 min at RT. Sections were incubated with a biotin‐conjugated secondary antibody followed by ABC (Vector Laboratories, Burlingame, CA, USA) reaction for 1 h at room temperature. Drosha signals were visualized using ImmPACT DAB solution (Vector Laboratories, Burlingame, CA, USA). The slices were counterstained with hematoxylin and dehydrated with graded ethanol solutions and xylene and mounted with Acrytol mounting medium (Leica, Wetzlar, Germany). Bright‐field images were acquired using a Nikon Optiphot‐2 microscope with Olympus cellSens Standard software. Quantification of positive cell number and the reciprocal DAB intensity was performed by Fiji software (ImageJ) using the protocol based on the method described before (Nguyen et al., 2013). Briefly, the maximum intensity value of an RGB image analyzed in ImageJ is 250. DAB staining exhibits an intensity less than 250, inversely correlating with the intensity of the staining. Reciprocal intensity yields from subtracting the intensity of the region of interest from 250, which is positively correlated with the intensity of the staining. The values obtained were analyzed by two‐tailed unpaired t test with GraphPad Prism (GraphPad Software, San Diego, CA, USA).

### 4.6 Preparation of tissue or cell extracts and western blot analysis

Brain tissues from human or rat were lysed with IP buffer containing 40 mM Tris‐HCl (pH 7.4), 150 mM NaCl, 0.5% sodium deoxycholate, 1 mM Na_3_VO_4_, 1% Triton X‐100 and EDTA‐free complete protease inhibitor and Phospho‐Stop inhibitor (Roche, Basel, Switzerland), and ultrasonic disruption. For examination of Triton soluble Drosha, the lysates were centrifuged at 11,000 rcf for 15 min at 4℃ and the supernatants were transferred to a new tube. For examination of Triton insoluble Drosha, the pellets were washed thoroughly with IP buffer and resuspended with urea lysis buffer containing 8 M urea, 40 mM Tris‐HCl (pH 7.4), 40 mM NaCl, 2 mM MgCl_2_, and EDTA‐free complete protease inhibitor and Phospho‐Stop inhibitor. The pellets were sonicated and centrifuged at 20,000 rcf for 10 min at 4℃. Protein concentrations were determined using the BCA protein assay kit (Thermo Fisher Scientific, Waltham, MA, USA). For Western blot analysis, equal amounts of protein samples were separated by sodium dodecyl sulfate‐polyacrylamide gel electrophoresis (SDS‐PAGE) and transferred to PVDF membranes (Bio‐Rad, Hercules, CA, USA), and the proteins were detected with indicated antibodies.

### 4.7 Cytoplasmic and nuclear fractionation

Cytoplasmic and nuclear fractionation was performed using EZ nuclei isolation kit (NUC101, Sigma‐Aldrich, St. Louis, MO, USA) according to the manufacturer's protocol. The nuclear fractions were washed thoroughly. Only the cytoplasmic fraction from the first lysate and the nuclear fraction from the last wash were used for Western blot analysis.

### 4.8 Solubilization of Aβ peptides and preparation of Aβ oligomers

The human Aβ 1‐42 peptide and Aβ 42‐1 peptide used in the present study was synthesized by Peptide 2.0 (Chantilly, VA, USA) with the purity of more than 95%. Lyophilized peptide was stored at −20℃ before dissolve. Prior to dissolve, the peptide was allowed to equilibrate to room temperature for 30 min. Firstly, the peptides were resuspended in 1,1,1,3,3,3‐hexafluoro‐2‐propanol (Sigma‐Aldrich, St. Louis, MO, USA) to 1 mg/ml and aliquoted into 1.5 ml microcentrifuge tubes. Then, the clear solution containing the dissolved peptide was allowed to dry under vacuum in a SpeedVac and stored at −20℃. The dried peptide was resuspended in dimethyl sulfoxide (Sigma‐Aldrich, St. Louis, MO, USA) to 5 mM by pipette mixing. Aβ oligomers were prepared by diluting dried peptide to 100 μM in phenol‐free F‐12 medium (Invitrogen, Carlsbad, CA, USA), vortexed for 30 s, and incubated at 4℃ for 24 h. The medium was centrifuged for 10 min at 4℃ at 14,000 *g*. The supernatant contained soluble Aβ oligomers was quantitated by BCA assay to 100 μM and verified by Western blot.

### 4.9 Dual luciferase reporter assay

Primary cortical neurons were seeded in 24‐well plate coated with poly‐l‐lysine. In 14 DIV, neurons were treated with 1 μM Aβ oligomers for 24 h before transfection. For luciferase reporter transfection, medium of every well were collected and stored at 37℃. Neurons were transfected with 100 ng per well of pri‐miRNA reporters with Neurobasal medium (without B‐27) using Lipofectamine LTX reagent (Invitrogen, Carlsbad, CA, USA) according to the manufacture's protocol. After 2 h, the transfect complex were discarded and the original medium were added back to each well for another 24 h. Firefly (FF) luciferase and *Renilla* (RL) luciferase were measured with Promega's dual‐luciferase kit and detected with a microplate reader (BioTek, Winooski, VT, USA).

### 4.10 Immunofluorescence

Human or rat brain slices were blocked with 5% goat serum and double stained with antibodies to Drosha and different cell‐specific markers, including NeuN, Iba1, GFAP, and Calbindin. Primary cortical neurons were seeded on the cover glasses pretreated with poly‐l‐lysine. In 14 DIV, neurons were treated with Aβ oligomers for the indicated time courses. The neurons were fixed with 4% paraformaldehyde and sequentially stained with Drosha and the secondary fluorescence‐conjugated antibodies. The cover glasses were mounted for observation. For neuronal transfection, neurons of 14 DIV were co‐transfected with GFP and pcDNA3, or GFP and pcDNA3‐Drosha plasmids for 12 h, and then, the neurons were treated with Aβ oligomers for another 48 h. Based on our previous study, during co‐transfection, if the labeled plasmid was successfully transfected then so was the second unlabeled plasmid (Mao et al., 1999). Neurons were stained with Hoechst (Thermo Fisher Scientific, Waltham, MA, USA) and fixed. Cover glasses were mounted and imaged with laser scanning confocal microscopy (Nikon, Minato, Tokyo, Japan).

### 4.11 Immunoprecipitation

Anti‐Drosha antibody was firstly incubated with neuronal lysate overnight at 4℃. After the lysate were washed three times with IP lysis buffer, the lysate was incubated with Dynabeads protein G (Thermo Fisher Scientific, Waltham, MA, USA) for another 4 h. The beads were washed with IP buffer, and the protein levels of phospho‐Ser were determined with the phospho S/P antibody by Western blotting.

### 4.12 Statistical analyses

Data were expressed as mean ± standard deviation (SD) from at least three independent experiments. Statistical analysis was carried out using GraphPad Prism (GraphPad Software, San Diego, CA, USA). Distribution of data was checked with the Shapiro–Wilk test or Kolmogorov–Smirnov test (*p* > 0.05) before analyzed with either two‐tailed unpaired t test or one‐way ANOVA followed by Tukey or Dunnett's t test. Differences were considered significant when *p* < 0.05.

## CONFLICTS OF INTEREST

The authors declare that they have no conflicts of interest with the contents of this article.

## AUTHOR CONTRIBUTIONS

H.X. and Z.M. designed the experiment and wrote the first draft of the manuscript. H.X., X.L., W.L., Y.X., P.S., B.M., and X.S. performed the experiments. B.T., Q.Y., and Z.M. analyzed the data. All authors read and approved the final manuscript.

## Supporting information

Fig S1

Fig S2

Fig S3

Table S1‐S3

## Data Availability

All data are in the manuscript and the associated supporting information file.
