# Supplementary figures and images for: p38 MAPK‐mediated loss of nuclear RNase III enzyme Drosha underlies amyloid beta‐induced neuronal stress in Alzheimer's disease
Source: Aging Cell. 2021 Sep 16;20(10):e13434. doi: 10.1111/acel.13434 (PMC8521488; doi:10.1111/acel.13434)

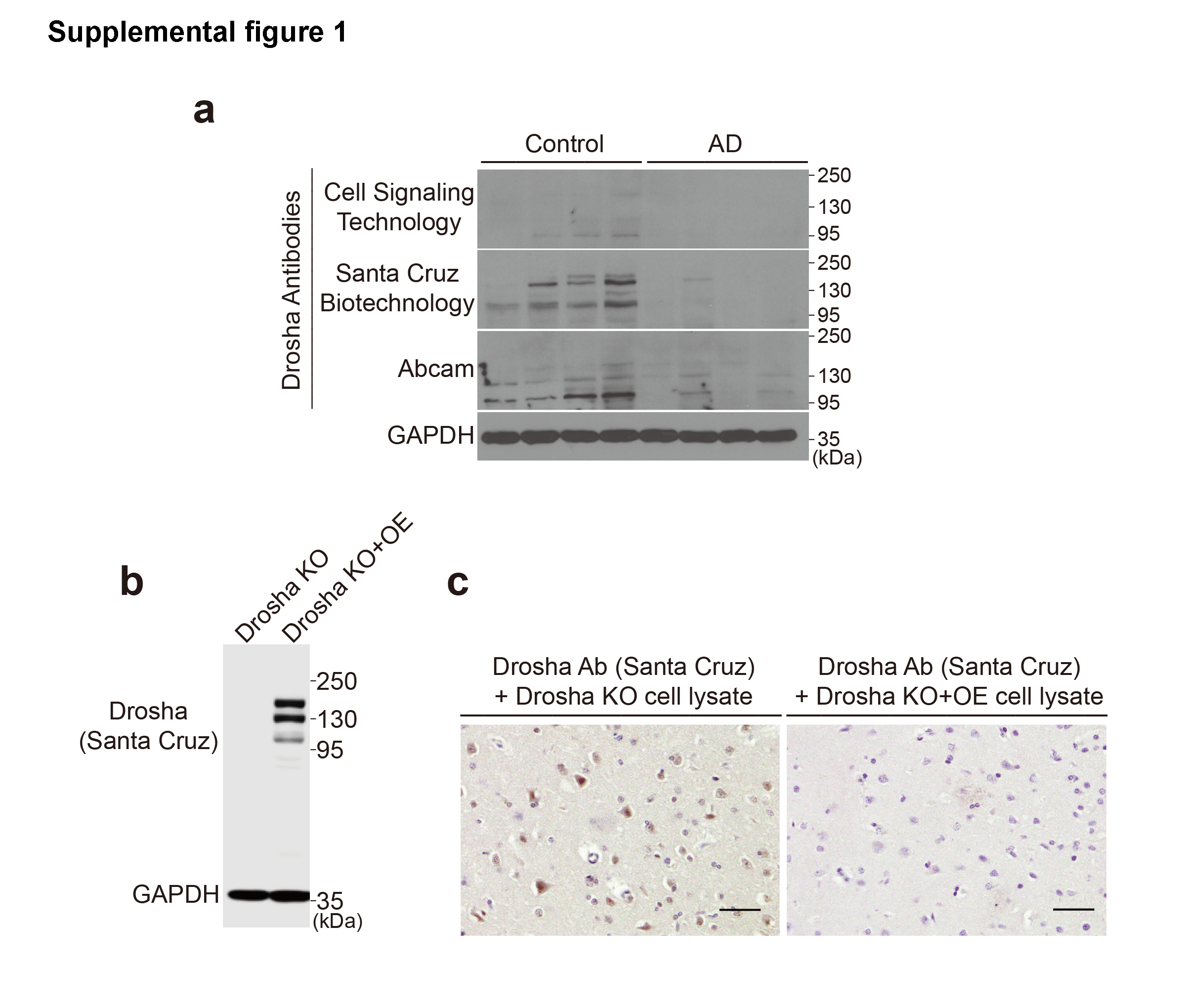

Supplement: Supplementary file 1 — Fig S1 [file ACEL-20-e13434-s003.png]

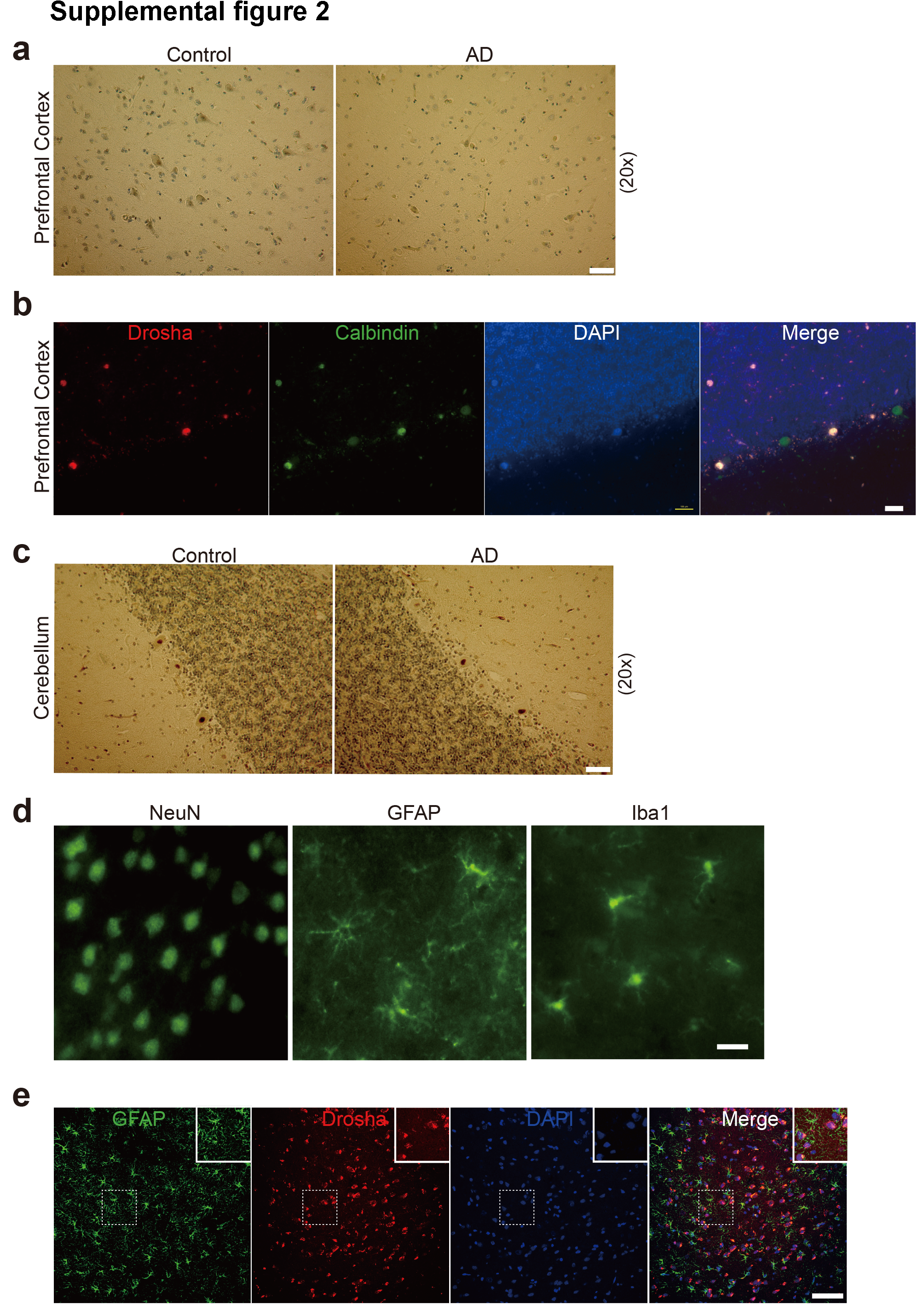

Supplement: Supplementary file 2 — Fig S2 [file ACEL-20-e13434-s001.png]

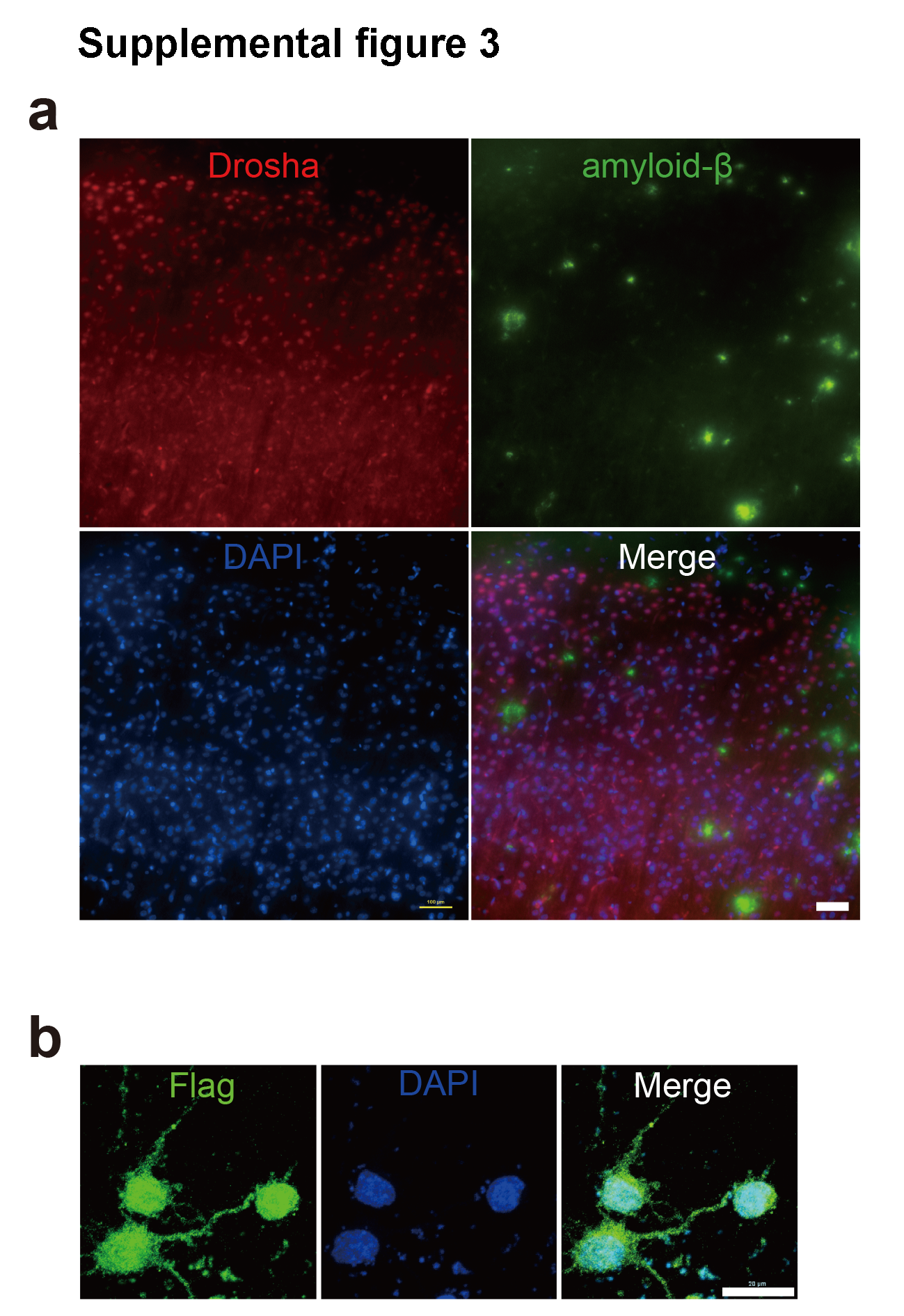

Supplement: Supplementary file 3 — Fig S3 [file ACEL-20-e13434-s002.png]
