# Supplementary material for: p38 MAPK‐mediated loss of nuclear RNase III enzyme Drosha underlies amyloid beta‐induced neuronal stress in Alzheimer's disease
Source: Aging Cell. 2021 Sep 16;20(10):e13434. doi: 10.1111/acel.13434 (PMC8521488; doi:10.1111/acel.13434)
Supplement: Supplementary file 4 — Table S1‐S3 [file ACEL-20-e13434-s004.docx]

**Supplemental table 1. Diagnostic information of human control and AD patients**

| **Case Number** | **Primary Neuropathologic Diagnosis** | **PMI (hr)** | **Age at Onset** | **Age at Death** | **Duration** | **ApoE** | **Race/Sex** |
| --- | --- | --- | --- | --- | --- | --- | --- |
| OS01-86 | Control | 22.5 |  | 51 |  | E2/3 | wm |
| OS00-06 | Control | 8 |  | 60 |  | E3/4 | bf |
| E05-74 | Control | 6 |  | 59 |  | E2/3 | bm |
| E08-137 | Control | 15.5 |  | 92 |  | E3/3 | wf |
| OS03-299 | Control | 6 |  | 69 |  | E3/3 | wm |
| OS03-394 | Control | 5 |  | 87 |  | E2/3 | wf |
| OS94-54 | Control | 10.5 |  | 96 |  | E3/3 | wf |
| E05-13 | AD | 5.5 | 83 | 88 | 5 | E3/3 | wm |
| E05-67 | AD | 11.5 | 52 | 62 | 10 | E3/4 | wm |
| E07-26 | AD | 4 | 74 | 85 | 11 | E3/4 | wm |
| OS00-05 | AD | 5 | 63 | 74 | 11 | E2/3 | wm |
| OS02-163 | AD | 11 | 53 | 70 | 17 | E3/4 | wm |
| OS97-54 | AD | 6 | 59 | 67 | 8 | E3/4 | wm |
| E07-84 | AD | 16 | 67 | 72 | 5 | E3/4 | wm |

Footnotes: AD, Alzheimer’s disease; PMI, post-mortem interval; ApoE, Apolipoprotein E; wm, white male; bf, black female; bm, black male; wf, white female.

**Supplemental table 2. Statistical analysis of human control and AD patients**

|  | Control | AD | *p* value |
| --- | --- | --- | --- |
| *n* | 7 | 7 |  |
| Age of death (yrs) | 73.43 ± 18.03 | 74.00 ± 9.40 | 0.9419 |
| PMI (hr) | 10.50 ± 6.40 | 8.43 ± 4.74 | 0.4958 |
| Duration (yrs) | - | 9.57 ± 4.16 | - |
| Gender (female/total) | 4/7 | 0/7 |  |

Footnotes: The numerical variables indicated the mean ± standard deviation. P values refer to analysis of two-tailed unpaired t test between control and AD groups.

**Supplemental table 3. Commercial anti-Drosha antibodies tested**

| Antibody name | Company | Catalog number |
| --- | --- | --- |
| RNase III Drosha antibody (C-7) | Santa Cruz Biotechnology | sc-393591 |
| Anti-Drosha antibody | Abcam | ab12286 |
| Drosha (D28B1) Rabbit mAb | Cell Signaling Technology | 3364 |
